# Pistachio Nuts (*Pistacia vera* L.): Production, Nutrients, Bioactives and Novel Health Effects

**DOI:** 10.3390/plants11010018

**Published:** 2021-12-22

**Authors:** Giuseppina Mandalari, Davide Barreca, Teresa Gervasi, Michael A. Roussell, Bob Klein, Mary Jo Feeney, Arianna Carughi

**Affiliations:** 1 Department of Chemical, Biological, Pharmaceutical and Environmental Science, University of Messina, 98166 Messina, Italy; davide.barreca@unime.it; 2 Department of Biomedical and Dental Sciences and Morphofunctional Imaging, University of Messina, 98125 Messina, Italy; teresa.gervasi@unime.it; 3 Janus Nutrition LLC, Rochester, NY 14618, USA; mike@mikeroussell.com; 4 California Pistachio Research Board, Fresno, CA 93727, USA; bobk@acpistachios.org; 5 Consultant to the Food and Agriculture Industries, Los Altos Hills, CA 94024, USA; mj@feeney.us.com; 6 American Pistachio Growers, Fresno, CA 93720, USA; arianna.carughi@gmail.com

**Keywords:** pistachio, polyphenols, health effects, flavonoids, nuts, production

## Abstract

Epidemiological and clinical studies have indicated positive outcomes related to tree nut consumption. Here, we review the production, nutrient, phytochemical composition and emerging research trends on the health benefits of pistachio nuts (*Pistacia vera* L.). Pistachios are a good source of protein, fiber, monounsaturated fatty acids, minerals and vitamins, as well as carotenoids, phenolic acids, flavonoids and anthocyanins. Polyphenols in pistachios are important contributors to the antioxidant and anti-inflammatory effect, as demonstrated in vitro and in vivo through animal studies and clinical trials. The antimicrobial and antiviral potential of pistachio polyphenols has also been assessed and could help overcome drug resistance. Pistachio consumption may play a role in cognitive function and has been associated with a positive modulation of the human gut microbiota and beneficial effects on skin health. Pistachio polyphenol extracts may affect enzymes involved in glucose regulation and so type 2 diabetes. Taken together, these data demonstrate the health benefits of including pistachios in the diet. Further studies are required to investigate the mechanisms involved.

## 1. Introduction

### 1.1. History

Members of the *Pistacia* genus belong to the Anacardiaceae family, which includes plants such as cashew nut, mango, sumac and poison ivy. *Pistacia vera* L. (pistachio) is the only species in this genus which produces edible nuts large enough to be commercially acceptable [1]. Other species are mainly used as rootstocks or for oil, agro-forestry, timber production and carpentry and include *P. atlantica, P. cabulica, P. chinensis, P. falcata, P. integerrima, P. kinjuk, P. kurdica. P. lentiscus, P. mutica, P. palaestina* and *P. terebinthus* [2]. There is controversy on the etymology of the word “Pistachio”. It probably derives from the word “pistak” in the ancient Persian language, Avestan [3]. Pistachios are native of western Asia and were distributed in the Middle East, Mediterranean countries and Europe by traders. Evidence of pistachio consumption dates back 300,000 years to the Neanderthals. Charred pistachio remains have been recovered from the Mousterian levels at Kebara Cave, Israel [4]. In modern history, remains of pistachio nuts dating from the sixth millennium BC have been found in both Afghanistan and southeastern Iran, where pistachio was likely first cultivated in regions close to where it grew wild. Pistachios were widely grown in the ancient Persian Empire, from where its cultivation gradually expanded to the West. Together with almonds, pistachios were the most popular nuts for human consumption and are the only two nuts mentioned in the Bible [5]. The Assyrians also used pistachios as medicines, aphrodisiacs and antidotes. Pistachios came late to Italy from Syria during the Roman era, circa the first century AD. It was referred to as the “Syrian nut”, and its price was exorbitant [5]. From Italy, pistachios spread into the Mediterranean regions of southern Europe and North Africa. Pistachios were imported by American traders in the 1880s primarily for United States (U.S.) citizens of Middle Eastern origin. Some 50 years later, and to the present, pistachios have become a popular snack [6]. Today, the U.S. is the world’s leading producer of pistachios, accounting in 2020 for approximately 47% of world production, followed by Turkey (30%) and Iran (19%) [7]. Commercial U.S. pistachio production takes place almost exclusively (99%) in California, with some production occurring in Arizona and New Mexico.

### 1.2. Production

The pistachio tree is deciduous and thrives in dry climates and poor soil conditions. *Pistacia* plants are very well adapted to desert and semi-desert areas of the temperate and sub-tropical regions. Although adapted to a wide range of different soil types, these plants prefer relatively deep, light or dry sandy loams with a high lime content. The growth response of pistachio plants to irrigation with hard water is excellent, and plants are tolerant to salinity in water and soil. A recent review of their efficiency of water use for cultivation shows that pistachio trees survive under drought conditions and that yield is not significantly affected by moderate and properly timed deficit-irrigation restrictions during the growing season [2]. This is noteworthy, as an aspect of sustainability that frequently arises in connection with agriculture is water usage, especially in semi-arid regions.

**Figure 1:**
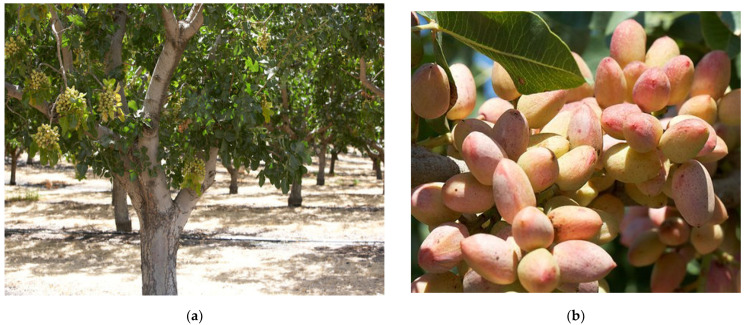
Fruit-bearing *Pistacia vera* L. Kerman cultivar (**a**); *Pistacia vera* L. ripe fruit (hull) (**b**).

**Figure 2:**
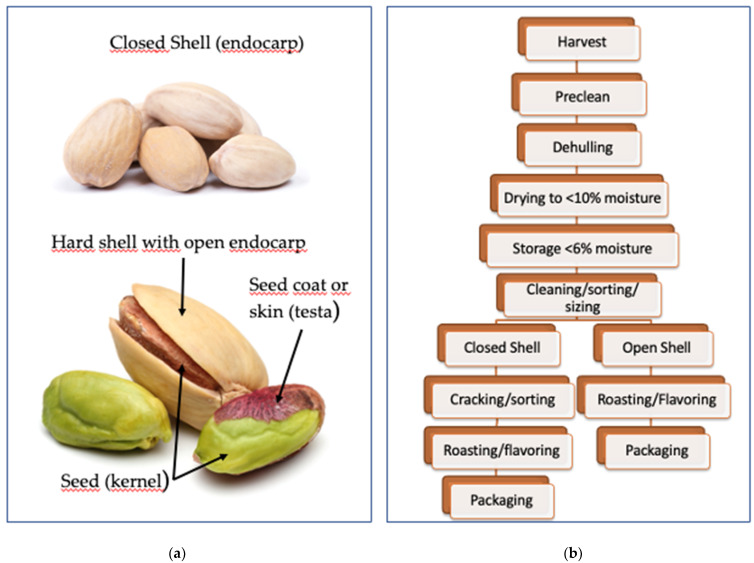
(**a**) Pistachio nuts (closed and open shell) illustrating their unique colors due to the presence of bioactive compounds, mainly lutein (yellow), anthocyanins (red) and chlorophyll (green); (**b**) pistachio processing: from harvest to consumer.

Pistachio trees are planted in orchards and take approximately 7 to 10 years to reach significant production (Figure 1a). They are “alternate bearing”, meaning that an entire tree alternates between a high production year followed by a year of low production. Peak production is reached around 15 years and can, with proper management, be maintained for decades subsequently. Californian pistachio orchards are expected to last for 70 to 100 years [8]. Trees grow to 10 m tall but are usually maintained at about 7 m. They are dioecious: both male and female pistachio trees are required for pistachio production, and usually 1 male tree is planted for every 8 to 24 female trees. The flowers do not have petals and are unisexual and borne in grape-like clusters. The fruits of the pistachio are drupe, containing edible single oval seeds, covered by a thin and soft seed coat (testa). The seeds are covered by an inedible, hard, smooth, cream-colored shell (endocarp) that is in turn covered by a fleshy, thin hull, pale green in color, with a blush of red color during maturity (Figure 1b). Seed color varies greatly from light to dark green or even greenish yellow, and it is composed of two cotyledons. The unique color of pistachio nuts is due to the combination of these beneficial compounds: the yellow color from catechins, lutein and zeaxanthin; the green from chlorophyll; and the purple outer seed coat from anthocyanins (Figure 2a). When the fruit ripens, the hull changes from green to an autumnal yellow-red. As shells reach full size, they begin to harden and then completely fill with the nut. As the nut enlarges, it causes the shell to split naturally. The splitting open is a trait that has been selected by growers and allows pistachios to be marketed largely in-shell for fresh consumption. Commercial cultivars vary in how consistently they split open.

The appropriate time of harvest is one of the most important factors affecting the quality of pistachios. In the U.S., pistachios are harvested starting in late August or early September by mechanically shaking the trees. Pistachios are subject to shell staining if they remain on the tree too long or if the hulls remain in contact with the shells for an extended period of time after harvest. Because of this, the industry concentrates its harvesting efforts for six to eight weeks during the harvest season (Figure 2b). At processing plants, hulls are removed using an abrasive peeler. Blank shells are separated from shells containing nuts. The nuts are first dried in vertical grain dryers for four to six hours to a moisture level of between 9 and 10%. They are then conveyed to silos where they are further dried with forced air to 5–7% moisture, generally taking 24 h for each percent reduction. The nuts can be stored in the silos until needed for further processing. Roasting pistachios is a conventional process in the nut industry to improve the aroma, texture, color, mouthfeel and consumer acceptance. Roasting is conducted after drying at higher temperatures for relatively short time periods [8]. Comprehensive reviews on pistachio production in Turkey, Iran and the U.S. have been published previously [9,10,11].

### 1.3. Cultivars

There are relatively a few pistachio cultivars that have been named and described. In California, pistachio production was almost entirely based on the female cultivar Kerman and the male cultivar Peter’s (Figure 1a). However, in 2002, the cultivars Golden Hills and Lost Hills were introduced and now make up over 95% of the orchards planted in California over the last 7 years [11]. Iran has more than 70 cultivars and genotypes of female pistachios, a large number of male genotypes and is one of the most important global sources of pistachio germplasm. The most famous types of pistachios in Iran are Koleghoochi, Akbari, Mumtaz, Badami Zarand, Sefid pistachio Nogh, Ahmad Aghaei, Ouhadi, Khanjari Damghan, Shah Pasand Damghan, and Qazvini pistachio. In Turkey, Antep, Kirmizi, Uzun, Halebi and Siirt are the major pistachio cultivars. Aegina is the main edible cultivar in Greece. The most common female cultivars in Australia are Kerman and Sirora. In Italy, there is historically a niche cultivation: The pistachios from Bronte are famous and are protected by the DOP (DOP stands for Denominazione d’Origine Protetta, in English this translates to Protected Designation of Origin) designation “Pistacchio Verde di Bronte”.

### 1.4. Nutrient and Bioactive Composition

**Figure 3:**
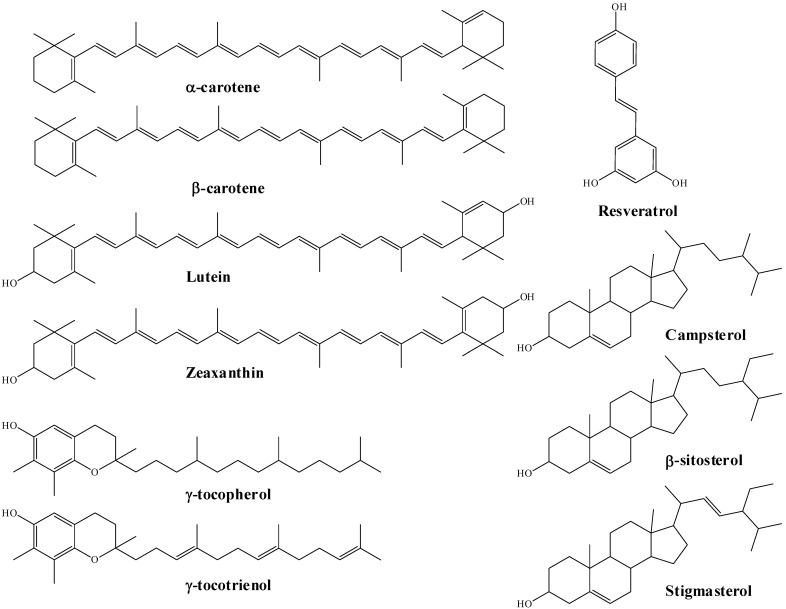
Chemical structures of the major bioactive carotenoids, steroids and resveratrol in pistachios.

Pistachios, like all nuts, have a high fat content, which is composed mainly of mono- and poly-unsaturated fatty acids as well as lower amounts of saturated fatty acids (Table 1). Of the fatty acids, oleic and linoleic acids represent more than half of the total pistachio fat content. Pistachios are also a good source of protein (about 21% of total weight). Roasted pistachios have a protein digestibility corrected amino acid score (PDCAAS) of 81, which drops to 73 when raw. The digestible indispensable amino acid score (DIAAS) values are 86 and 83 for raw and roasted pistachio nuts, respectively [12]. Pistachios have an essential amino acid ratio higher than most other commonly consumed nuts, with a high percentage of branched chain amino acids [12]. Pistachios are a good source of fiber, having 10% by weight of insoluble fiber and 0.3% of soluble fiber (Table 1). Pistachios are a source of at least 15 different micronutrients in significant amounts, based on the U.S. Food and Drug Administration (FDA) (providing over 10 % of the Daily Recommended Value (DRV) per ounce (28.5 g) serving) or the European Union (EU) (providing at least 15% Nutrient Reference Value per 100 g) Nutrition and Health Claims Regulation (NHCR) thresholds [13,14]. As such, pistachios are a significant source of protein, fiber, copper, manganese, vitamin B6, thiamin, potassium, phosphorous, chromium, vitamins E and K (phylloquinone), riboflavin, folate, magnesium, iron, zinc and selenium (Table 2), with smaller quantities of other micronutrients also present. Moreover, pistachios are also a rich source of lutein and zeaxanthin (xanthophyll carotenoids) and a broad range of bioactive phenolic compounds (Table 3, Figure 3).

With their long history of acceptance and use in different cultures and cuisines, pistachio nutrient profiles and bioactive attributes contribute to diet quality and can help populations lower their risk of contemporary health conditions associated with energy-dense, low nutrient quality diets. Health and nutrition guidance recommendations encourage a change to more plant-based food choices, including a shift within the protein category; research has moved away from focusing on total fat to the discovery of benefits of different types of fatty acids; and consumers are interested in the functional benefits associated with bioactive compounds. This accumulating evidence supports the inclusion of pistachios in nutritious dietary patterns that support a healthy lifestyle [26,27,28].

**Figure 4:**
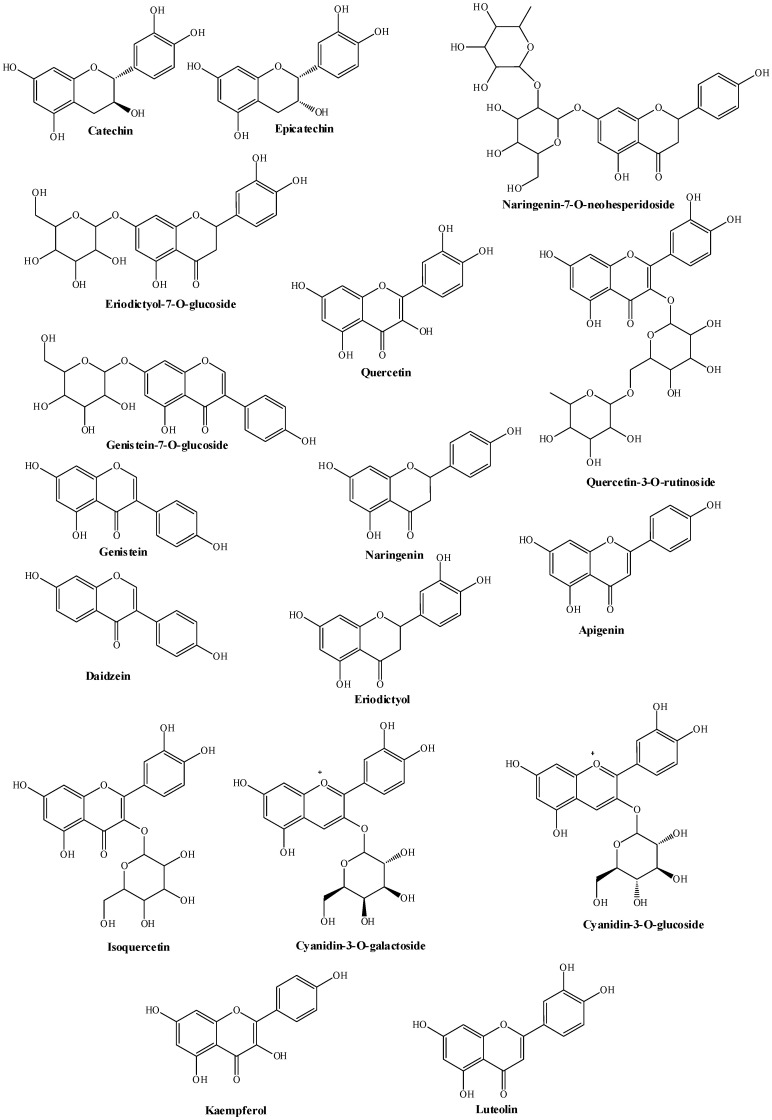
Chemical structures of pistachio flavonoids.

The main class of pistachio phenolic compounds include the flavonoids. Flavonoids have the basic skeletal structure of C6-C3-C6, and depending on their degree of oxidation and substitution on the 3-position, can be further divided into flavones, flavonoids, flavanols (e.g., catechin, epicatechin) and flavanones (e.g., quercetin, kaempferol, luteolin, apigenin), isoflavones (e.g., genistein, daidzein) and anthocyanidins. The latter, the anthocyanidins, are water-soluble pigments which occur naturally as glycosides (Figure 4).

Particular attention should be paid to the many flavonoid compounds of pistachios due to the type, the quantity and the availability of these bioactive compounds. The main flavonoids identified in pistachios are flavanols, flavonols, flavanones, isoflavones and anthocyanins, reaching 16–70 mg/100 g depending on the variety [19]. Among nuts, pistachios are the richest source of isoflavones, with reported values as high as ~3.63 mg/100 g (mainly as genistein-7-*O*-glucoside, genistein and daidzein) [29]. The flavonoid distribution and predominance differ between the skin and the kernel. Pistachio kernels are characterized by the presence of catechin, eriodictyol-7-*O*-glucoside, genistein-7-*O*-glucoside, naringenin-7-*O*-neohesperidoside, rutin (quercetin-3-*O*-rutinoside), isoquercetin, genistein, eriodictyol, daidzein and apigenin [30]. Pistachio skins contain these flavonoids with the exception of the isoflavones and apigenin. Pistachio skins also contain epicatechin, quercetin, naringenin, luteolin, kaempferol and the anthocyanidins (cyanidin-3-*O*-galactoside and cyanidin-3-O-glucoside) [30,31]. A mixture of anacardic acid homologues represent the main component of pistachio hulls, followed by fatty acids, phytosterols, carotenoids, chlorophylls, tocopherols and three triterpene acids (mangiferolic, isomangiferolic and mangiferonic acids) [32]. Raw pistachios are a source of proanthocyanidins, also known as condensed tannins, oligomeric and polymeric end-products of the flavonoid biosynthetic pathway. The identified compounds include dimers, trimers, tetra-esamers and epta-decamers with a total amount of ~258 mg/100 g [25]. They represent one of the final frontiers in flavonoid research, with a large body of literature emerging over the past few years, describing the potential health promoting properties of monomeric and polymeric flavan-3-ol derivatives, thought responsible of the so-called “French Paradox” [33]. Pistachio polyphenols, without differences between raw or roasted pistachios, are bioaccessible in the stomach and small intestine and therefore available for absorption, thus supporting a possible beneficial relationship between pistachio consumption and health-related outcomes [34,35]. Flavonols and flavanones are, among the flavonoid’s subclasses, perhaps the most studied compounds due to their health promoting properties identified through epidemiological studies, and theiranti-aggregative, antioxidant, cardioprotective, antibacterial, antiviral and anticancer activities have been reported [35,36]. Anthocyanidins are able to bind metals due to the presence of an *O*-diphenol group in the basic chemical structure. This is important in the inhibition of metal-induced lipid oxidation and contributes significantly to the antioxidant potential of pistachio flavonoids [37]. Gentile et al. showed that some of the health promoting properties of pistachios can be attributed partially to the content of the nut’s dietary antioxidants [23]. The effects of a hydrophilic extract of *P. vera* L. on the production of reactive oxygen species (ROS) in RAW 264.7 macrophage cells were evaluated in vitro. A dose-dependent decrease in the production of lipopolysaccharide-induced ROS in the presence of different concentrations of hydrophilic extract was observed, supporting the evidence that proanthocyanidins can be the bioactive components responsible for the effects on ROS [32]. Ballistrieri et al. have evaluated the effect of the ripening and drying process on polyphenols and tocopherols content of unpeeled *Pistacia vera* L. var. *bianca*. The results showed that the anthocyanin content increased with ripening, but sun-drying led to a substantial loss. Flavonoids and tocopherols decreased both with ripening and drying [38].

## 2. Antioxidant Potential of Pistachios In Vitro and In Vivo

Due to their high content of bioactive compounds, pistachio nuts represent a rich source of antioxidants including tocopherols, phylloquinone, carotenoids, chlorophyll and flavonoids. These constituents support pistachios being among the top 50 foods in total antioxidant capacity [30]. However, the amounts can vary significantly depending on genotype, pre- and post-harvest and storage conditions and the different measures of antioxidant activity [39].

### 2.1. In Vitro Studies

Several in vitro and in vivo studies have investigated the antioxidant effects of pistachio flavonoids by comparing the activity of the different parts of the nut or of different extracts (i.e., lipophilic or hydrophilic). Polyphenols and thus flavonoids are widely present in all portions of the pistachio nut. Lipophylic antioxidants (carotenoids and tocopherols and chlorophyll) are predominant in the kernel [23]. By using the [2,2′-Azinobis(3-ethylbenzothiazoline-6-sulfonic acid)] diammonium salt (ABTS) radical cation decolorization test, Gentile et al. showed that the hydrophilic extract of pistachio had a higher total antioxidant activity than the lipophilic one. The antioxidant activity of the hydrophilic fraction also inhibited, in a dose-dependent manner, both the metal-dependent and -independent lipid oxidation of bovine liver microsomes and the Cu2^+^-induced oxidation of human low-density lipoprotein (LDL).

In vitro tests such as the 2,2-diphenyl-1-picrylhydrazyl (DPPH) assay, the Folin–Ciocalteau colorimetric method, the Trolox equivalent antioxidant capacity (TEAC) assay and the superoxide dismutase (SOD)-mimetic assay have been used to compare the antioxidant capacity of pistachio kernel and skin. Tomaino et al. showed that pistachio skins had a higher antioxidant activity and a higher content of antioxidant phenolic compounds than the kernels [24]. To evaluate the differential contribution of pistachio skin and kernel extracts to in vitro antioxidant activity, Grace et al. incubated lipophylic and hydrophilic extracts with lipopolysaccharide (LPS)-activated RAW 264.7 macrophages [32]. All extracts significantly reduced the generation of ROS, with the skin’s hydrophilic extract showing the highest inhibition. This extract was also the most effective in reducing nitric oxide (NO) production, while lipophilic extracts (skin and kernel) were less effective. Since the skin polar extract had the highest phenolic content, the authors concluded that the antioxidant activity is connected to the polyphenolic constituents. Pistachio hulls, considered as agricultural waste, also have been shown to contain high antioxidant properties by evaluating the oxidation of soybean oil after a heating treatment, followed by peroxide value and the thiobarbituric acid value determinations [40]. Barreca et al. also reported on the antioxidant activity of pistachio hull extracts as measured by the DPPH assay; the superoxide anion scavenging assay (O⋅_2^−^_); the ferric reducing antioxidant power (FRAP); the Trolox equivalent antioxidant capacity (TEAC) assay; the oxygen radical absorbance capacity (ORAC assay); and the lipid peroxidation assay [41]. Grace et al. conducted an ROS assay and a NO radical inhibition assay to confirm the antioxidant potential of pistachio hulls [31]. Furthermore, pistachio (*Pistacia vera* L. variety Bronte) hull essential oil demonstrates antioxidant and free radical scavenging properties. Smeriglio et al. highlighted these properties by using the hydrogen atom transfer- and electron transfer-based methods and concluded that these are mainly attributable to the high amount of monoterpene hydrocarbons [42].

### 2.2. Animal Studies

Paterniti et al. compared the antioxidant activity of polyphenol extracts from natural raw shelled pistachios (NP) with those of roasted salted pistachios (RP) using an in vivo model of paw edema in rats [43]. The formation of nitrated proteins was blocked only in rats treated with NP. These results demonstrate that polyphenols present in pistachios possess antioxidant activity and agree with the study conducted by Gentile et al. (above), which reported that roasting results in a 60% reduction of the total antioxidant activity [23]. Aksoy et al. fed rats either 20 or 40% of their energy in the form of pistachios for 10 weeks. A significant increase in the activities of paraoxonase 1 (PON1) and arylesterase, both markers of antioxidant capacity, was observed in both groups supplemented with pistachios compared with the control (no pistachios) [44].

### 2.3. Clinical Studies

Clinical studies have shown that pistachio consumption results in a positive influence on oxidation biomarkers and antioxidant defenses. In a randomized, controlled, cross-over feeding trial on 28 hypercholesterolemic adults, Kay et al. showed that the consumption of diets containing 10 and 20% of energy from pistachios increased the serum concentration of γ-tocopherol, lutein and β-carotene and decreased the concentrations of oxidized LDL [45]. In a parallel-design study, 44 healthy individuals were randomized to a regular nut-free diet group or a pistachio group, consisting of 20% of their daily energy intake, for three weeks. The pistachio diet increased blood antioxidant potential by the production of thiobarbituric acid-reactive substances and decreased levels of malondialdehyde level (MDA) [46]. In a prospective study, Sari et al. fed a Mediterranean diet to 32 healthy young men. After four weeks, pistachios were added to the diet by replacing the monounsaturated fat content constituting approximately 20% of daily caloric intake [47]. Pistachios significantly decreased the total oxidant status, lipid hydroperoxide and malondialdehyde and increased levels of superoxide dismutase. These studies indicate that pistachio consumption may reduce oxidative stress markers in healthy subjects. Hernandez-Alonso et al., over an 8-week period, randomized 54 prediabetic individuals into 2 different crossover sequences, consisting of a pistachio-supplemented diet (57 g pistachio/day) and a control diet. During the pistachio diet intervention, oxidized-LDL decreased significantly and increased during the control diet period [48]. A recent systematic review and meta-analysis of 11 RCTs found that pistachio consumption significantly reduced MDA levels [49].

Thus, these studies indicate that pistachios have an antioxidant function that contributes to the health benefits associated with their consumption (Table 4). Pistachios are a complex whole food containing an array of bioactive compounds such as carotenoids, polyphenols, phylloquinone and tocopherols. Their antioxidant activity is most probably due to the synergistic action of many components. The inclusion of foods rich in these health-protective bioactive compounds in the diet can increase the quality of life and may influence the aging process.

## 3. Anti-Inflammatory Potential of Pistachios In Vitro and In Vivo

Systemic chronic inflammation is known to be an underlying causal factor in the progression of chronic conditions such as type 2 diabetes and metabolic syndrome. Pistachios contain a wide variety of nutrients and bioactive components, which may moderate inflammation. While the anti-inflammatory activities of bioactive compounds in pistachios have been investigated in vitro and in animal models, a limited number of studies, however, have been carried out using pistachios.

### 3.1. In Vitro Studies

Various in vitro LPS-stimulated cell models have been used to investigate the anti-inflammatory activities of pistachios in both skin and kernel. An in vitro LPS-stimulated murine RAW 264.7 macrophage model has been used to investigate the anti-inflammatory activities of pistachio skin and kernel by the characterization of the common genetic biomarkers associated with inflammation, such as interleukin 6 (IL6), inducible nitric oxide synthase (iNOS) and cyclooxgenase 2 (COX2) and by assessing their effects on mitochondrial bioenergetics and oxidative burst. Phenol-rich hydrophilic extracts of both pistachio skin and kernel inhibited the expression of the three tested pro-inflammatory markers, IL6, iNOS and COX-2 with the highest activity against the COX-2 gene. The polar pistachio skin extract had the greatest effect on the reduction of non-mitochondrial oxidative burst associated with inflammatory response in macrophages [32]. Paterniti et al. (described above) also evaluated the anti-inflammatory effect of a pistachio polyphenol extract using an LPS-stimulated monocyte/macrophage cell-line J774-A1 model. They found that NP slightly reduced the degradation of inhibitors of nuclear kappa B (IκB-α,), and both NP and RP reduced the tumor necrosis factor alpha (TNF-α) and interleukin 1 beta (IL-1β) production in a dose-dependent way [43].

### 3.2. Animal Studies

Different in vivo models have been used to investigate the anti-inflammatory effect of pistachio nuts and their components. In particular, the carrageenan-induced paw edema model was used to investigate the beneficial effects of the polyphenol rich extracts from pistachio nuts. A histological evaluation showed that the administration of NP resulted in a significant reduction in myeloperoxidase (MPO) activity, an amelioration of histological damage and a reduction in the infiltrating inflammatory cells [43]. The modulative activity of pistachio polyphenols in the inflammatory process was reported using a myocardial ischemia-reperfusion (MI-R) injury model in diabetic rats. Treatment with NP reduced myocardial tissue injury, neutrophil infiltration, adhesion molecules (ICAM-1, P-selectin) expression, proinflammatory cytokine (TNF-α, IL-1β) production, nitrotyrosine and poly(ADP-ribose) formation, NF-κB expression and apoptosis (Bax, Bcl-2) activation [50].

### 3.3. Clinical Studies

The anti-inflammatory effects of pistachios also have been reported in several clinical trials by assessing changes in biomarkers of inflammation such as C-reactive protein (CRP), tumor necrosis factor alpha (TNF-α) and the adhesion molecules (ICAM-1 and VCAM-1). In a 24-week controlled-feeding trial, 60 individuals with metabolic syndrome were randomized to either a pistachio group (corresponding to 20% of total energy) or a control group. Pistachio supplementation promoted improved inflammatory parameters, such as high-sensitivity CRP and interleukin-6 (IL-6) [51]. In the prospective study by Sari et al. mentioned above, pistachios significantly decreased serum IL-6, but not CRP or TNF-α levels [47]. In the study by Hernandez-Alonso, described above, the pistachio intervention reduced levels of fibrinogen and platelet factor 4 but not IL-6. However, IL-6 mRNA and resistin gene expression in lymphocytes decreased significantly [48]. Parham et al., in a double-blind, placebo-controlled trial, randomized 44 prediabetic individuals into 2 different crossover sequences, feeding a snack of 25 g of pistachio nuts twice a day for 12 weeks or a control pistachio-free snack. Although the overall changes in CRP due to the pistachio intervention were not significant, eating pistachios significantly reduced CRP levels when the intervention sequences were analyzed separately [52]. Sauder randomized 30 adults with well-controlled type 2 diabetes in a crossover, controlled feeding study. After a two-week run-in period, participants consumed diets with pistachios (contributing 20% of total energy) or without pistachios for four weeks each, separated by a two-week washout. Levels of CRP, ICAM, VCAM and e-selectin did not change significantly [53]. 

A recent meta study on 13 randomized controlled trials assessed the effect of pistachios on inflammatory markers CRP and TNF-α. Pistachio consumption did not have a significant effect on either [54]. These studies provide some evidence of the beneficial effects of pistachios on inflammation, although the results are inconsistent (Table 4). Systemic chronic inflammation may represent an important factor in the etiology of chronic diseases such as type II diabetes, cardiovascular diseases and metabolic syndrome over a person’s life span. The lack of consistency in findings relating to existing markers of inflammation suggests a need for more research in this area.

## 4. Emerging Research Trends in Pistachio Health Effects

Previous and present research has examined pistachios’ macronutrient content in areas, such as weight management and lowering the risk of chronic disease related to obesity, such as cardiovascular disease and glycemic control [55,56]. Systematic reviews and meta-analyses of RCTs suggest beneficial effects on blood pressure, endothelial function and on markers of glucose and insulin metabolism [49,54,57,58]. These have been compiled in excellent reviews [26,27,59]. Emerging research seeks to discover benefits related to the many bioactive compounds present in pistachios that have antioxidant and anti-inflammatory properties. These include neurocognition, the regulation of the gut microbiota and other quality of life conditions. Discussed in the following paragraph is an effort to broaden awareness of these recent and novel research topics. As we discuss them below, it is recognized that the paper does not provide a comprehensive research review of all pistachio health effects.

### 4.1. Pistachios and Cognitive Function

Emerging evidence suggests the beneficial role of flavonoids in cognitive performance [60,61,62]. Flavonoids, known to be powerful antioxidants and to have anti-inflammatory activity, are promising agents to lower the risk of memory and cognitive decline [63,64,65] given the role of oxidative stress and chronic inflammation in these age-related conditions. In a recent study, the long-term intake of dietary flavonoids was shown to be associated with lower odds of subjective cognitive decline in U.S. men and women [66].

Pistachios are rich in flavonoids such as anthocyanins and flavones and fat-soluble antioxidants such as tocopherols and carotenoids that may be related to beneficial effects on cognitive function. Thus far, pistachios’ benefits against the neurodegeneration induced by a high-fat diet in a mouse model have been investigated. The supplementation of pistachios in a high-fat diet prevents obesity-related neurodegeneration and reduces lipid dysmetabolism, oxidative stress, and mitochondrial dysfunction [67]. Other animal studies have evaluated the efficacy of pistachio supplementation in improving memory and motor function. Pistachios inhibited cognitive and motor damage and reversed spatial memory disturbances caused by two neurotoxic anticancer drugs, cisplatin and vincristine [68]. Haider et al., using a model of rats with rotenone-induced Parkinson’s disease, showed the positive effects of pistachios on neurobehavioral and neurochemical modifications, highlighting their neuroprotective effects and ability to improve memory and motor deficits [69]. A pistachio ethanolic extract, at a concentration of 200 and 400 mg/kg, led to cognitive improvements in Swiss Albino mice, using experimental memory impairments induced by scopolamine [70].

Various studies have shown the potential of pistachios in reducing anxiety-like behavior, using an extract administered at a concentration of 10 and 100 mg/kg in ovariectomized rats [71] and in a study using a hydroalcoholic extract of ripe pistachio hulls at a concentration of 10 mg/kg dose in the elevated plus maze model of anxiety [72]. The ovariectomized mice study also demonstrated pistachios’ efficacy in increasing working memory and physical power [71]. The Y-maze Continuous Alternation Task and the Morris Water Maze tests in Wistar rats evaluated the effect of *Pistacia vera* seed oil on working memory and on spatial learning and memory, respectively. Pistachio oil improved memory and cognitive impairment, but it had no effect on spatial memory parameters [73].

Other bioactive compounds of pistachios contributing to cognitive health are the xanthophyll carotenoids lutein and zeaxanthin. These dietary carotenoids can pass over the blood–brain barrier and are selectively incorporated in the macular region of the retina and the brain. In the macula, lutein and zeaxanthin are referred to as macular pigment (MP). In the brain, they account for 66–77% of the total carotenoid brain content, implying a role in cognition [74]. Using MP density as a biomarker of brain xanthophyll concentration a relationship between these carotenoids and temporal processing speed has been suggested, which is a necessary component of sensory and cognitive functions such as language, executive function, learning and memory. Lutein and zeaxanthin have also been found to improve gap junctional communication, which is necessary for light processing and the development of neural circuitry in the visual system. These are also related to an increase in visual processing speed [74]. The effect of pistachio consumption, on MP lutein/zeaxanthin content and cognitive function has not been studied, yet it is a promising area. Compared to green leafy vegetables, lutein content in pistachios is low, but its bioavailability is higher, enhanced by the presence of fatty acids.

Taken together, these results suggest a potential beneficial effect of pistachios and pistachio extracts in cognitive function; however, further preclinical studies should be performed to understand the mechanisms involved. While research continues, a high adherence to a Mediterranean style diet has been associated with slower cognitive decline, with reduced risk of mild cognitive impairment conversion to Alzheimer’s Disease (AD) and with reduced risk of AD [75]. This dietary pattern is characterized by abundant plant food consumption, including nuts.

### 4.2. Pistachios and Gut Microbiota

The human gut microbiota is a complex microbial ecosystem whose composition varies across the digestive tract, the large bowel being the most densely populated. Bacteria, archaea and eukarya of the human gut microbiota create mutually beneficial relationships affecting the host’s general condition during homeostasis and disease [76,77,78]. Dysbiosis, defined as a disruption to the microbiota homeostasis caused by an imbalance in the microflora, changes in their functional composition and metabolic activities or a shift in their local distribution, may be related to the pathogenesis of type 2 diabetes, obesity, cardio-metabolic diseases, non-alcoholic fatty liver disease and malnutrition [79,80]. Diet is considered one of the main drivers in shaping the gut microbiota. Two systematic reviews reported recently on the effect of tree nuts on the adult human gut microbiota and gut function [81,82]. The effect of pistachio consumption on the gut microbiota composition has been reported [83]. The microbiota composition collected from volunteers, recruited to participate in two separate randomized, controlled, crossover studies assigned to three treatment groups (no nuts; 42.5 g/d either almonds or pistachios and 85 g servings/d of either almonds or pistachios) was analyzed. The effect of pistachio consumption on gut microbiota composition was stronger than almond consumption, with an increase in the number of beneficial butyrate-producing bacteria. Furthermore, a decrease in lactic acid bacteria was demonstrated after pistachio consumption, whereas *Bifidobacterium* numbers were not affected by the consumption of either almonds or pistachios. A randomized crossover study assessed whether the chronic consumption of pistachios was able to modify the urine metabolome in prediabetic subjects: The results showed that certain urinary metabolites related to the gut microbiota metabolism and the tricarboxylic acid cycle, associated with insulin resistance and type 2 diabetes, were significantly modulated [84].

Animal studies also assessed the effect of pistachios on the gut microbiota in diabetic rats and in mice fed a high-fat diet. Yanni et al. demonstrated that a pistachio-based diet for four weeks was able to restore normal microbiota and enhanced the number of beneficial bacteria (such as *Bifidobacterium*, *Lactobacillus*, *Turicibacter* and *Romboutsia*) in rats with streptozotocin-induced diabetes [85]. Terzo et al. administered a high-fat diet supplemented with pistachios for 16 weeks to 8 rats with previously induced inflammation and dysbiosis [28]. The results showed that pistachio consumption decreased inflammation in obese mice and determined positive effects on the gut microbiota’s composition, with an increase in the numbers of *Parabacteroides, Dorea, Allobaculum, Turicibacter, Lactobacillus* and *Anaeroplasma* and a reduction in the number of bacteria associated with inflammation, such as *Oscillospira, Desulfovibrio, Coprobacillus* and *Bilophila.*

Taken together, data from clinical and animal studies confirm pistachio consumption may yield a modulatory effect on the gut microbiota. However, further investigations are required to establish variations in trial design and microbiota inter-variability. 

### 4.3. Effects of Pistachio Flavonoid Extracts on Diabetes Related Enzymes

Diabetes impacts 1 of 11 people worldwide and is projected to increase by over 40% to 700M people by 2045 [86]. Type 2 diabetes is a chronic metabolic condition that impacts glucose homeostasis via several different underlying mechanisms but manifests itself in the form of prolonged hyperglycemia. A systematic review found that 50–57 g of pistachios daily for 1–4 months yields significant improvements in fasting glucose, fasting insulin, homeostatic model assessment for insulin resistance (HOMA-IR) and fructosamine [87].

The effect of pistachios in glycemic control is often attributed to a combinatory effect of being a low-carbohydrate food that contains fat, fiber and protein—all of which can slow gastric emptying rate and thus postprandial glycemia. However, the flavonoid content of pistachios is often overlooked with respects to the mechanism behind the anti-diabetic effects of pistachios. Pistachio flavonoids can beneficially impact several enzymes that regulate glucose control and homeostasis.

Alpha-glucosidase (α -glucosidase) is an enzyme found on the brush border of the small intestine responsible for the cleavage of the alpha bonds, breaking down starches to glucose. Alpha-glucosidase inhibitors are a widely used class of oral anti-diabetes medications, taken with meals and used to slow the breakdown of starches and disaccharides to glucose, attenuating the rate in which glucose can be absorbed in the small intestine [88]. Pistachio flavonoids, quercetin-3-O-rutinoside, genistein, isoquercetin, rutin and quercetin are all known inhibitors of α -glucosidase [89] and are potentially more effective when present as a mixture of more than one flavonoid, as found in foods [90]. Promising preliminary studies have looked at taking advantage of the α -glucosidase inhibition of pistachio flavonoids by including pistachio hull and peel extracts into pasta and ice cream, respectively, to obtain more food choices for diabetic individuals [91,92].

Pistachio flavonoids also impact enzymes responsible for glucose metabolism and homeostasis. Babujanarthanam et al. reported that the administration of quercetin for 30 days to streptozotocin-diabetes-induced rats led to the reversal of enzymatic activity observed with the onset of chemically induced diabetes [93]. Quercetin led to increased hexokinase, decreased glucose-6-phosphase and decreased fructose-6-bisphosphate activities. These changes improved the overall glucose homeostasis in the diabetic rats. Prince et al. reported similar improvements in hexokinase, decreased glucose-6-phosphase and decreased fructose-6-bisphosphate activity after 45 days of rutin (quercetin-3-O-rutinoside) administration to diabetic rats [94]. In in vitro models, rutin can increase glucose transporter type 4 action via MAP (mitogen-activated protein) kinase and PI3K (atypical protein kinase C), suggesting an insulin-mimetic for rutin, as well as positively impacting (GLUT-4) translocation [95,96].

Collectively, the preliminary mechanistic studies suggest that pistachio flavonoids have direct positive action towards enzymes that regulate carbohydrate digestion, metabolism and homeostasis. More research needs to be conducted in translating these early-stage findings into clinical studies to examine the role of pistachio flavonoids in the treatment and prevention of type 2 diabetes.

### 4.4. Pistachios and Photoprotective Effect

Several studies have demonstrated the potential of plant antioxidants to protect animals from alterations induced by ROS overproduction, including skin damage caused by exposure to ultraviolet (UV) radiation [97,98]. The antioxidant properties and the in vivo photoprotective effect of pistachio (Bronte variety) seed and skin extracts have been evaluated [99]. Radical scavenger/antioxidant properties were exhibited, mainly in the skin extract, which was more abundant in anthocyanins. The same extract was able to reduce, when topically applied, UVB-induced skin erythema in human volunteers at a higher degree compared to the seed extract. A more recent study employing a three-dimensional human skin equivalent tissue model established a beneficial effect of pistachio (Kemin variety) bioactives on skin health and aging [64]. The authors demonstrated that pistachio antioxidants preserved skin thickness and fibroblast morphology in human skin equivalent exposed to UVA irradiation. Furthermore, lutein and γ-tocopherol protected against changes to the epithelial and connective tissue prior to UVA exposure.

These findings suggest that pistachio phenolic-rich extracts could be successfully employed as photoprotective ingredients in cosmetic and pharmaceutical formulations for topical use.

### 4.5. The Antimicrobial and Antiviral Potential of Pistachio Polyphenols

The increased rates of drug resistance pose a serious treat, and global effort is focused on the discovery of novel therapeutics with antimicrobial and antiviral effect to be used either alone or in combination with existing drugs. It is well accepted that plant extracts represent an important source of bioactive compounds which may be used for their antimicrobial and antiviral activity [100]. Previous research demonstrated that polyphenol-rich extracts obtained from raw shelled and roasted salted pistachios were active against a range of Gram-positive bacteria, with a bactericidal effect observed against *Listeria monocytogenes* (both ATCC strains and food isolates), *Staphylococcus aureus* and methicillin-resistant *Staphylococcus aureus* (MRSA) clinical isolates [101]. Furthermore, clinical isolates of *Staphylococcus* spp. were CHARACTERIZED and tested for their sensitivity against polyphenol-rich extracts from pistachios. The data confirmed that both the natural raw and the roasted salted fractions were active against *S. aureus* 6538P and *Staph.* spp. clinical isolates [102]. Another study confirmed the activity of a methanolic extract of *Pistacia vera* against staphylococcal infections [103]. Pistachio polyphenols could therefore be used to identify novel therapeutics for the treatment of *S. aureus* skin infections.

Recently, Di Lodovico et al. evaluated the potential of *Pistacia vera* L. oleoresin against resistant *Helicobacter pylori* strains in combination with levofloxacin [104]. The results showed a protective effect against *H. pylori* infection using an in vivo model of *Galleria mellonella* (62% and 63% of survival using oleoresin and levofloxacin, respectively). The antibacterial, anti-virulence and anti-biofilm activities of *Pistacia vera* L. oleoresin were also evaluated against oral streptococci, including *Streptococcus mutans* [105]. The essential oil from *Pistacia vera* L. variety Bronte hull was bactericidal against a range of *S. aureus* strains as well as the Gram-negative strain of *Escherichia coli* at the concentration of 7.11 mg/mL, whereas no effect was obtained on *Pseudomonas aeruginosa* ATCC 9027 [42]. The same essential oil was fungicidal at concentrations between 2.50 and 5.0 mg/mL against a number of standard and clinical strains of *Candida* sp. Specifically, D-limonene and 3-Carene were identified as the components with major activity [106]. Recently, a pistachio hull extract was used as a reducing and stabilizing agent in the preparation of copper nanoparticles with antibacterial potential against Gram-positive and Gram-negative strains and antifungal effect against *Aspergillus niger* [107].

The antiviral potential of pistachio polyphenols also has been explored against herpes simplex virus type 1 (HSV-1) replication. The esults demonstrated that treatment with a natural raw pistachio polyphenol-rich extract reduced the expression of the viral proteins ICP8 (infected cell polypeptide 8), UL42 (unique long UL42 DNA polymerase processivity factor) and US11 (unique short US11 protein) and determined a decrease in viral DNA synthesis. Furthermore, the anti-herpetic effect was confirmed using a mix of pure polyphenol compounds present in the extract [108].

These findings suggest that the antimicrobial and antiviral effects of pistachio extracts are the result of a balance of the individual bioactive compounds that in combination exert the activity. Therefore, pistachio polyphenols, alone or in association with existing drugs, could be considered good candidates for the development of novel topical formulations. Further studies are warranted to evaluate the mechanisms of action involved in the observed effect.

### 4.6. Pistachios and Retinal Health and Disease

Lutein and zeaxanthin are present in high concentrations in the retina, particularly in the macula. They work as a filter protecting the macula from blue light and also as a resident antioxidant and free radical scavenger to reduce oxidative stress-induced damage [109]. Many observational and interventional studies have suggested that lutein and zeaxanthin may reduce the risk of various eye diseases, especially late forms of age-related macular degeneration. In vitro and in vivo studies indicate that they could protect the retina against oxidative damage and so lower risk of disease progression. While studies have looked at the effect of supplementing the diet with lutein-rich foods such as eggs, dark leafy vegetables and avocados on MP density, this yet has to be explored with pistachios [110,111].

## 5. Conclusions

Pistachios have been part of our diet since prehistoric times and have a long history of acceptance and use in different populations around the world. There is scientific consensus that pistachio consumption has a beneficial impact on human health. They are nutrient-dense and rich in bioactive compounds which play a crucial role on their health effects. The present review article summarized pistachio bioactives, particularly phenolic compounds, as important contributors to their antioxidant and anti-inflammatory effects. Emerging research is also reviewed showing that pistachio consumption may play a role in cognitive function, in modulating the human gut microbiota and may have beneficial effects on the skin and the health of the retina. Pistachio polyphenol extracts may affect enzymes involved in glucose regulation and thus type 2 diabetes. Finally, pistachio polyphenols have antimicrobial and antiviral potential. This accumulating evidence supports the inclusion of pistachios in nutritious dietary patterns that support a healthy lifestyle and the aging process. Because of their nutrient profile, pistachios are a tasty and versatile alternative to many foods. They are consumed as healthy snacks and can be added to many savory dishes as well as topping for salads, yogurts and dips. Their practical advantages could benefit different population groups, contributing to a healthy, balanced, more plant-based diet. In addition, pistachios can be viewed as a promising candidate to address the need for both nutrition and sustainable agricultural practices. Therefore, studies could be performed to establish the mechanisms related to pistachio’s beneficial health effects.

## Figures and Tables

**Table 1:** Pistachio (raw, unsalted) composition.

Macronutrient and Energy Content	g/100 g
Protein	20.2
Total lipid (fat)	45.3
Saturated fatty acids	5.9
Monounsaturated fatty acids	23.3
Polyunsaturated fatty acids	14.4
Carbohydrate, by difference	27.2
Fiber, total dietary	10.6
Sugars, total	7.66
Starch	1.67
Energy	2340 kJ

Source: U.S. Department of Agriculture Food Data Central 2019 [15].

**Table 2:** Pistachio (raw, unsalted) micronutrient content.

Minerals	Percentage (mg/100 g)	Vitamins	Percentage (mg/100 g)
Calcium	105	Vitamin C, total ascorbic acid	5.6
Iron	3.92	Thiamin	0.87
Magnesium	121	Riboflavin	0.16
Phosphorous	490	Niacin	1.3
Potassium	1020	Pantothenic acid	0.52
Sodium	1	Vitamin B-6	1.7
Zinc	2.2	Folate, total	51
Copper	1.3	Vitamin B-12	0
Manganese	1.2	Vitamin A	514IU
Selenium	0.007	Vitamin E (alpha-tocopherol)	2.86
Fluoride,	0.0034	Vitamin D (D2 + D3)	0
		Vitamin K (phylloquinone)	0.0013 *

Source: U.S. Department of Agriculture Food Data Central 2019 [15]; * Popa, S. et al. [16].

**Table 3:** Bioactive compounds in pistachios.

Compound, Unit	Percentage (mg/100 g) g	Seed Part
Carotenoids, total		Kernel
Beta-carotene	0.305 [15]	
Alpha-carotene	0.010 [15]	
Lutein + Zeaxanthin	2.9 [15]	
Chlorophylls		Kernel (different varieties)
Chlorophyll a	1.8 to 15.0 [17,18]	
Chlorophyll b	1–5.0 [17,18]	
Pheaphytin a	2.6 [18]	(Bronte)
Gamma-tocopherol	20.6	Kernel
Gamma-tocotrienol	1.67	Kernel
Phytosterols, total		Kernel
Campsterol	10 [15]	
Beta-sitosterol	198 [15]	
Stigmasterol	5 [15]	
Phenolics, total, mg GAE	1677 [19]–1420 [20]	Skin and kernels
Resveratrol	0.006–0.697 [21,22,23]	Kernel
Flavonoids	16–70 [19,24]	Skin and kernels
Anthocyanins (cyanidin-3-galactoside, cyanidin-3-glucoside)	69.6 [6]	Skin
Proanthocyanidins	211–307 [25]	Skin and kernels
Isoflavones (genistein, genistein-7-o-glucoside, dadzein)	159 [26]	Kernels

**Table 4:** Antioxidant and anti-inflammatory effects related to pistachio consumption.

Study Design	Study Population	Outcome	Reference
Animal study	Rats with paw edema induced by carrageenan	↓ nitrated proteins ↓ myeloperoxidase (MPO) activity, ↓histological damage ↓the infiltrating inflammatory cells	Paterniti, 2017 [43]
Animal study	Rats	↑ paraoxonase 1 (PON1) ↑ arylesterase	Aksoy, 2007 [44]
Animal study	Diabetic rats with myocardial ischemia-reperfusion (MI-R) injury	↓ myocardial tissue injury ↓ neutrophil infiltration, ↓adhesion molecules (ICAM-1, P-selectin) expression, ↓ proinflammatory cytokine (TNF-α, IL-1β) production↓ nitrotyrosine and poly(ADP-ribose) formation, ↓NF-κB expression and apoptosis (Bax, Bcl-2) activation	Di Paola, 2018 [50]
Randomized, controlled, cross-over	Hypercholesterolemic adults	↑ γ-tocopherol, ↑lutein ↑ β-carotene ↓ oxidized LDL	Kay, 2010 [45]
Parallel-design study	Healthy individuals	↑ blood antioxidant potential (thiobarbituric acid-reactive substances) ↓ malondialdehyde level (MDA)	Kocyigit, 2006 [46]
Prospective study	Healthy young men	↑ superoxide dismutase ↓total oxidant status ↓ lipid hydroperoxide ↓malondialdehyde ↓serum IL-6	Sari, 2010 [47]
Crossover clinical trial	Prediabetic individuals	↓oxidized-LDL ↓ fibrinogen levels ↓platelet factor ↓IL-6 mRNA and resistin gene expression in lymphocytes	Hernandez-Alonso, 2014 [48]
Systematic review and meta-analysis of 13 randomized controlled trials		↓MDA levels No significant effect on CRP and TNF-α.	Ghanavati, 2010 [49]
Controlled-feeding trial	Individuals with metabolic syndrome	Improved inflammatory parameters, such as high-sensitivity CRP, and interleukin-6 (IL-6)	Gulati, 2014 [51]
Double blind, placebo-controlled trial	Prediabetic individuals	↓CRP levels	Parham, 2014 [52]
Crossover, controlled feeding study	Adults with well-controlled type 2 diabetes	Levels of CRP, ICAM, VCAM and e-selectin did not change significantly	Sauder, 2015 [53]

## Data Availability

Not applicable.
